# The Design of a Glycerol Concentration Sensor Based on an LRSPP Hybrid Photonic Biosensor

**DOI:** 10.3390/s23042010

**Published:** 2023-02-10

**Authors:** Magno M. de Araújo, José P. da Silva

**Affiliations:** 1 Electrotechnical Department, Federal Institute of Education, Science and Technology of Rio Grande do Norte, Mossoro 59628-330, Brazil; 2 Post-Graduated Program in Electrical and Computer Engineering, Technology Center, Federal University of Rio Grande do Norte, Natal 59078-970, Brazil

**Keywords:** surface plasmon polariton, LRSPP, refractive index sensor, on-chip, optical sensor

## Abstract

A refractive index sensor based on an on-chip silicon nitride (Si_3_N_4_) ridge waveguide long-range surface plasmon polariton (LRSPP) is theoretically designed. The waveguide sensor consists of a gold film to enable the plasmonic resonance on top of a Cytop polymer layer. A proper finite element method was used to design and optimize the geometric parameters at the optical wavelength of 633 nm. In addition, the spectral performance was evaluated using the transfer matrix method from 580 to 680 nm. The redshifted interference spectrum results from an increasing analyte refractive index. The sensitivities of 6313 dB/cm/RIU and 251.82 nm/RIU can be obtained with a 400 nm wide and 25 nm thick Au layer. The proposed sensor has the potential for point-of-care applications considering its compactness and simplicity of construction.

## 1. Introduction

On-chip silicon photonic circuits present a feasible technology to guarantee the development of the pharmaceutical, healthcare, and food industries [1,2]. For example, in the medical field, photonic biosensors could provide real-time diagnosis of diseases and personalized clinical monitoring and treatment, embedded in portable and automated systems, as expected for point-of-care (POC) diagnostics [3,4].

Glycerol is one of the most versatile components of the food and pharmaceutical industries, acting as a solvent for flavors and food color or in the production of cough syrups, expectorants, and cardiac medications, for instance [5]. Furthermore, in the biofuel industry, glycerol is the main byproduct of biodiesel production. As a result of its ample occurrence in nature, many microorganisms can bioconvert glycerol into an energy and carbon source, which adds considerable value to the biodiesel industry. In addition, biotechnology allows microorganisms to convert glycerol into some petroleum-related chemical commodities, reinforcing the biodegradable polymer production chain [6]. Moreover, when glycerol is used as a carbon source, other microbial products such as medicinal drugs, antibiotics, and fine chemicals can be obtained. Therefore, real-time monitoring of glycerol concentrations in aqueous solutions is essential to guarantee the quality of the final products in those industries [7].

Nowadays, the gold-standard method for biomarker detection is the enzyme-linked immunosorbent assay (ELISA), which needs specialized reagents, has a bulky setup, and has a long preparation time [8]. However, fast and economical techniques are desired to meet the requirements of POC applications, such as food safety analysis [9]. As a result, label-free photonic biosensors can meet these requirements.

Silicon photonic biosensors depend on waveguide nearinfrared light confinement to detect refractive index (RI) changes in their sensing regions. The light’s portion that travels outside the waveguide interacts with the surrounding analyte, changing the guided light behavior [10]. Therefore, monitoring the properties of the output light, such as the emission, adsorption, transmission, light scattering, and polarimetry, can enable the detection of the analyte of interest [8,11].

Several silicon photonic structures have been designed as label-free biosensors, covering different optical measurements, for example, interferometer-based [12,13], ring resonators [14,15], Bragg grating resonators [16,17], and photonic crystals structures [18,19]. However, the most well-known label-free optical biosensors have been surface plasmon resonance (SPR)-based since Biacore commercialized the first model in 1990 [20,21].

SPR is the collective oscillation of free electrons between the interface of two materials with dielectric functions of opposite signs when induced by an incoming light [22]. For instance, when a transverse magnetic (TM) polarized light excites a metal–dielectric interface, surface plasmon polaritons (SPP) propagate and have a rapidly decaying evanescent field in the dielectric layer [23]. A different number of methods can be applied to excite the SPPs, including prism coupling [24,25], grating coupling [26,27,28], and waveguide coupling [29,30].

In a structure with a metal film between two dielectrics, surface polariton modes occur on both sides of the film, with fields decaying away from the metal–dielectric interface. That configuration supports bound and leaky SPP modes with symmetric and antisymmetric TM fields across the metal layer [22]. The symmetric SPP mode shows the longest propagation distance and the lowest propagation losses, known as the long-range SPP (LRSPP). In the other case, the antisymmetric mode presents a shorter propagation distance and the highest losses, termed short-range SPP (SRSPP). For a metal layer with adequate thickness, in addition to the LRSPP and SRSPP modes, the metal–dielectric structure supports a hybrid mode, a combination of the dielectric and SPP modes [30,31]. The LRSPP presents an attenuation two to three times lower than the single interface SPP, which results in a longer propagation distance [32].

To guarantee the LRSPP mode symmetry, the difference between the dielectrics’ RI on each side of the metal layer should be minimum. Therefore, low-RI polymers are usually employed as cladding materials. For example, Teflon and Cytop present the RI close to the water in aqueous solutions. On the other hand, for analytes with RI over 1.5, the ultraviolet-cured polymer SU-8 works better [31]. 

In this paper, an LRSPP hybrid sensor based on a strip waveguide operating at 633 nm for glycerol concentration measurement is theoretically analyzed. First, Section 2 presents the sensor structure and materials. Moreover, the simulation method to obtain the propagation parameters is described in Section 3. Furthermore, Section 4 explains the design methodology and performance parameters applied. Then, Section 5 investigates the spectral performance of the propagation length, propagation loss, and transmittance. In addition, the bulk and spectral sensitivity for the LRSPP waveguide sensor is calculated.

## 2. Sensor Structure

**Figure 1:**
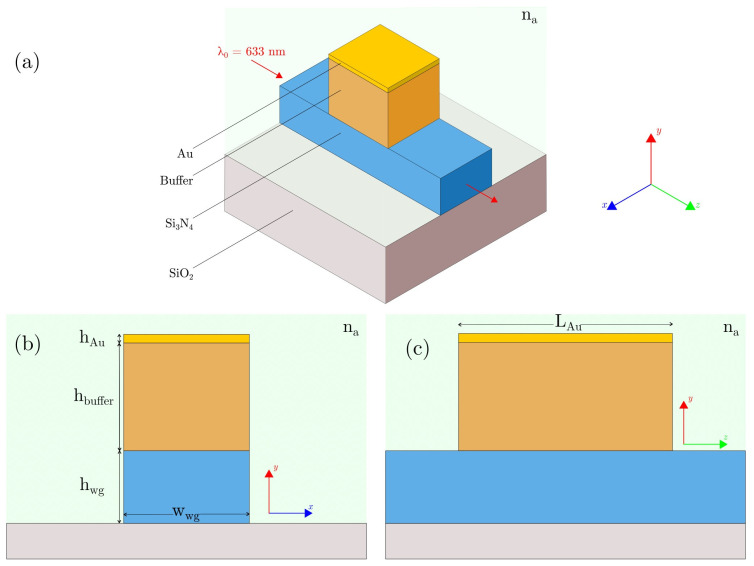
(**a**) Conceptual schematic of the long-range surface plasmon polariton (LRSPP) on-chip waveguide sensor. (**b**) Cross-sectional dimensions for the sensor. (**c**) Longitudinal dimensions for the Cytop and *Au* layers.

The on-chip proposed LRSPP sensor consists of a ridge waveguide of Si_3_N_4_ on top of a SiO_2_ substrate. Above a session of the waveguide are a Cytop buffer and a *Au* layer, as shown in Figure 1a, to detect changes in the analyte’s refractive index (n_a_).

The proposed structure is based on the compatibility of Si_3_N_4_ with the complementary metal–oxide semiconductor (CMOS) technology. Furthermore, silicon nitride presents a lower refractive index than silicon in aqueous analytes. In addition, this technology presents low loss and low thermal sensitivity, which is crucial to biosensing applications [33]

Figure 1b shows the cross-section view of the sensor, in which the waveguide has width w_wg_ and height h_wg_, and the Cytop layer and the Au layer have heights h_buffer_ and h_Au_, respectively. In Figure 1c, the length L_Au_ is the same for the Cytop and *Au* layers. The SiO_2_ substrate thickness is 2 μm.

The analyte is a glycerol solution in water of refractive index n_a_, which ranges from 1.33 to 1.3574 [34], as shown in Table 1. The light in the mid-infrared and infrared ranges presents strong absorption in aqueous media, which can obscure analyte signals [23]. Thus, the propagation parameters analysis was performed in part of the visible light spectrum. Therefore, the structure design applied a wavelength of 633 nm, red light, to define the sensor’s dimensions. At λ_0_ = 633 nm, the refractive index of the Si_3_N_4_, SiO_2_, Cytop, and Au were n_core_ = 2.04, n_c_ = 1.4507 [35], n_buffer_ = 1.34 [36], and n_Au_ = 0.3114 + j3.1405 [37], respectively.

The proximity between the analyte and Cytop refractive index values improves the LRSPP mode, increasing the propagation length and reducing the propagation losses [36]. 

## 3. Structure Simulation

The design of an LRSPP sensor needs the determination of the optical propagation properties, e.g., the complex effective refractive index (n_eff_), electric and magnetic field distribution, propagation loss, and sensing length. These characteristics were numerically determined using a suitable finite element method (FEM) [38,39]. In this method, the wave equation is specified in the magnetic field transversal components, in which its divergent is set to zero to eliminate the spurious modes. The Galerkin method discretizes the wave equations in the form $\left[A\right]\frac{\partial \left\{{\vec{h}}_{T}\right\}\left(z\right)}{\partial z}=\left[B\right]\left\{{\vec{h}}_{T}\right\}\left(z\right)$, in which [A] and [B] are matrices that contain the geometry function and electromagnetic parameters of the structure, and $\left\{{\vec{h}}_{T}\right\}\left(z\right)$ is the transversal magnetic field component. That differential equation system is solved using the Crank–Nicholson finite difference method in the z-direction. Therefore, the FEM method described was implemented using the FORTRAN language. A mesh generation software created the triangular mesh, which was exported to the FORTRAN program. Moreover, a perfectly matched layer (PML) was applied to limit the computational domain. 

Another characteristic to assist in the sensor’s design is the spectral response. The transfer matrix method (TMM) is the proper tool to describe that response [40]. 

Since each device section has its *n_eff_*, it can be analyzed as an optical multilayer structure [41]. Defining two fundamental wave matrices, one for the propagation in a uniform segment and another for propagation between interfaces, Equations (1) and (2), respectively, it was possible to obtain the total transfer matrix for the whole waveguide. In Equation (1), i is the section number, $\varphi =n_{eff}k_{o}d$, $k_{o}$ is the vacuum wavenumber, and d is the section length; in Equation (2), $n_{i}$ is the effective refractive index of the i-th section. 

Equation (3) shows the total transfer matrix for this work structure, which relates the amplitudes of the forward and backward waves at the input and output ports. Therefore, using Equations (4) and (5), the transmission (t) and reflection (r) coefficients were extracted [40].

(1) $$M_{i}=\left[\begin{array}{cc} e^{-j\varphi } & 0\\ 0 & e^{j\varphi } \end{array}\right]$$

(2) $$M_{i\left(i+1\right)}=\frac{1}{2n_{\left(i+1\right)}}\;\left[\begin{array}{cc} n_{\left(i+1\right)}+n_{i} & n_{\left(i+1\right)}-n_{i}\\ n_{\left(i+1\right)}-n_{i} & n_{\left(i+1\right)}+n_{i} \end{array}\right]\;$$

(3) $$M_{tot}=\left[\begin{array}{cc} A & B\\ C & D \end{array}\right]=M_{3}M_{23}M_{2}M_{12}M_{1}\;$$

(4) $$t=\frac{AD-BC}{D}\;$$

(5) $$r=-\frac{C}{D}\;$$

**Figure 2:**
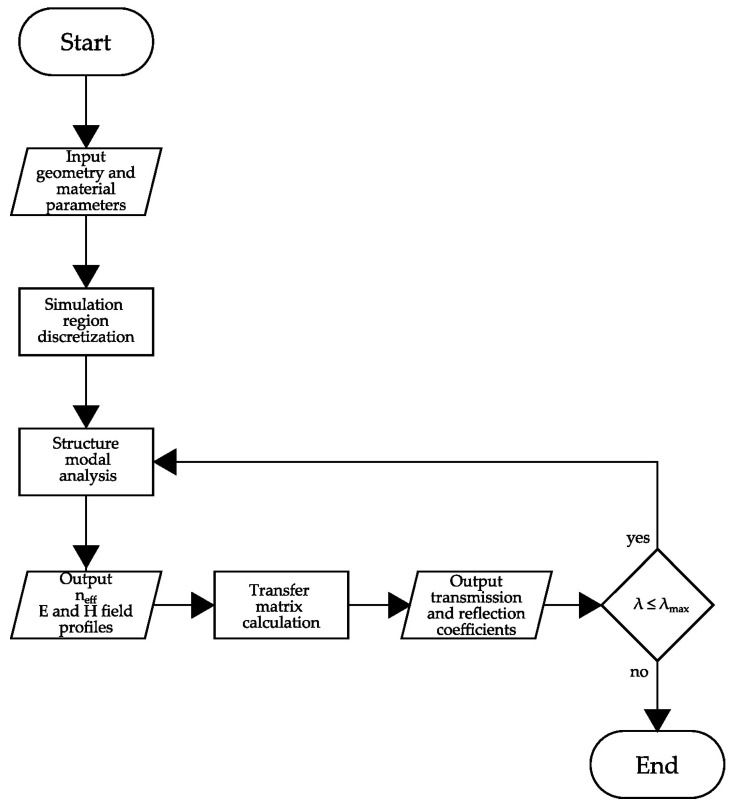
Flowchart to obtain the device parameters.

The TMM computation above was implemented in a MATLAB® algorithm. Then, the simulation was repeated for each wavelength to plot the transmission and reflection spectrums. It is essential to note that the n_eff_ depends on the wavelength, which must be considered in the computation. Hence, the numerical FEM calculated the n_eff_ in the range of 580 nm to 680 nm for each waveguide section. Figure 2 presents a flowchart with the mentioned steps.

## 4. Design and Optimization

The waveguide dimensions were chosen based on single-mode propagation. Then, a parametrical analysis was performed to find that region of propagation. The h_wg_, λ_0,_ and n_a_ were fixed at 200 nm, 633 nm, and 1.33, respectively, and the w_wg_ was changed from 100 to 500 nm. Moreover, the h_buffer_ was studied in the range from 30 to 500 nm.

**Figure 3:**
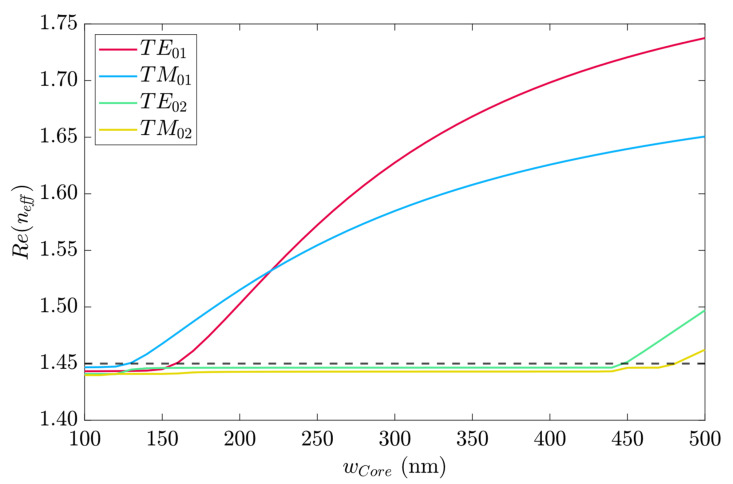
*Re*(*n_eff_*) for the first four fundamental modes of the 220 × 350 nm Si_3_N_4_ waveguide.

Figure 3 shows the real part of the effective refractive index (n_eff_) for the first four fundamental modes for the simulated parameters. The dashed line indicates the limit under which there was no propagation. Hence, from w_wg_ = 130 to w_wg_ = 160 nm, only the quasi-TM_01_ mode propagated. From that point, the quasi-TE_01_ mode transmitted, and until w_wg_ = 450 nm, only these two modes propagated. Hence, to guarantee the quasi-TM mode propagation, the chip would be excited by end-fire coupling with a polarization-maintaining fiber, for example [31].

To avoid multiple quasi-TM modes guiding and to allow the highest power transmission, the width w_wg_ = 400 nm was used for the subsequent analysis.

**Figure 4:**
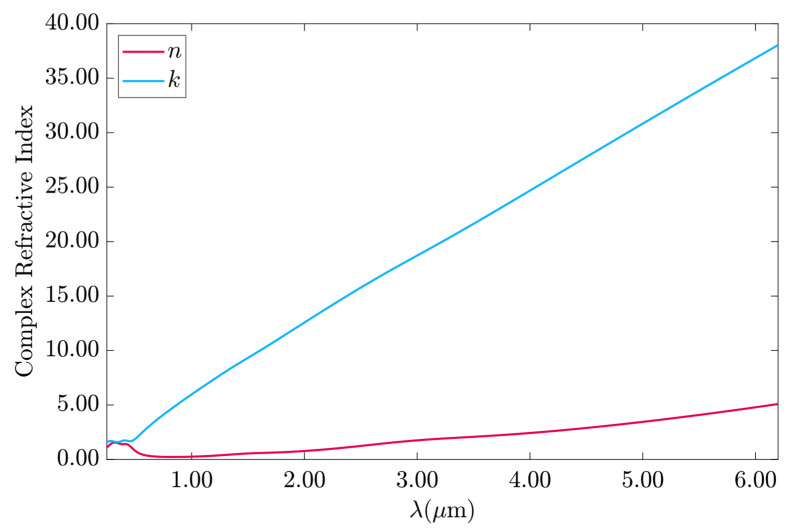
The complex refractive index of gold from the Drude–Lorentz model.

The complex refractive index of the metal layer was obtained from the Drude–Lorentz model, shown in Figure 4, using Equation (6) and converted to a refractive index with the relation $n=\sqrt{\epsilon }$. The parameters used in are Equation (6) are listed in Table 2 [37]. At λ_0_ = 633 nm, the gold complex refractive index is 0.3114 + j3.1405.

In Equation (6) $\tilde{\epsilon }$ is the complex relative permittivity, *ω_p_* is the plasma frequency, *i* is the number of oscillators with frequency $\omega_{p}$ and strength $f_{i}$, and Γ*_i_* is the damping rate.

(6) $$\tilde{\epsilon }\left(\omega \right)=1+\omega_{p}^{2}\overset{5}{\underset{0}{\sum }}\frac{f_{i}}{\omega_{i}^{2}-\omega^{2}-i\omega \Gamma_{i}}$$

The behavior of the plasmonic effect depended on the metal layer thickness. For example, two independent plasmonic modes occurred at both gold sides when its thickness was larger than the skin depth. In the other case, when the metal layer was thinner than the skin depth, these two modes interacted and originated the hybrid modes: dielectric-LRSPP and dielectric-SRSPP. With the decrease in the metal layer beyond the skin depth, the SRSPP attenuation and effective mode index increased, while the same parameters in the LRSPP mode decreased [42].

**Figure 5:**
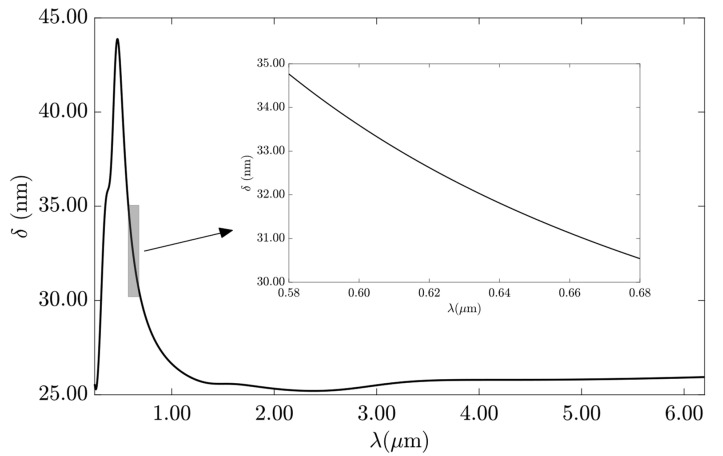
The skin depth penetration for gold at the optical frequency range. Inset: closeup of the region from 0.58 to 0.68 μm.

Figure 5 shows the skin depth penetration for gold, from 0.25 to 6.1 μm, obtained from Equation (7) [43], in which *k* is the imaginary part of the Au complex refractive index. Gold had the highest current penetration at 0.470 μm with 43.88 nm of skin depth. In the range of 0.58 to 0.68 μm, the skin depth varied from 34.77 to 30.54 nm, respectively. For λ_0_ = 633 nm, the skin depth was δ = 32.08 nm.

(7) $$\delta \left(\lambda \right)=\frac{\lambda }{2\pi k}\left(m\right)$$

The gold thickness must be lower than 30.54 nm for the LRSPP hybrid mode to propagate in that frequency range. Therefore, the suitable h_Au_ was evaluated using the propagation length (PL) and propagation loss (α), as shown in Equations (8) and (9) [44], respectively, in the range from 15 to 35 nm.

(8) $$PL=-\frac{\lambda_{0}}{4\pi k}\;\left(m\right)$$

(9) $$\alpha =-\frac{0.4\pi k}{\lambda_{0}}\log\left(e\right)\;\;\left(dB/cm\right)$$

**Figure 6:**
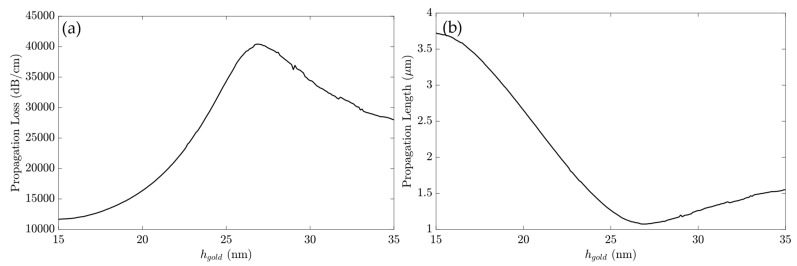
The gold layer above the waveguide. (**a**) α versus h_Au_ and (**b**) PL versus h_Au_.

Figure 6 shows the PL and α for the gold layer directly applied to the waveguide, in which the thickness of the gold was analyzed in the propagation parameters. 

As shown in Figure 6a, the propagation loss of the SPP with the gold layer directly above the waveguide had its maximum at h_Au_ = 26.8 nm, with 40420 dB/cm. The propagation length, presented in Figure 6b, had its maximum at h_Au_ = 15 nm, with 3.72 μm, and its minimum at h_Au_ = 27 nm, reaching 1.08 μm. The following analysis used h_gold_ = 25 nm to retain a strong field confinement around the gold layer, indicated for the propagation loss value of 34299 dB/cm.

The Cytop layer, known as the buffer layer, with an n_buffer_ = 1.34, decreased the refractive index gradient between the dielectric surrounding the gold layer, improving the LRSPP characteristics [36].

**Figure 7:**
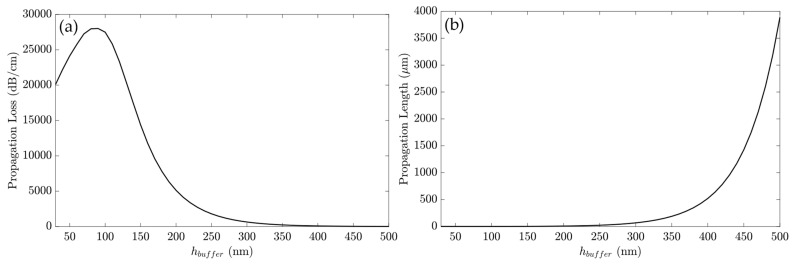
The Cytop layer between the waveguide and the gold layer. (**a**) α versus h_buffer_ and (**b**) PL versus h_buffer_.

A parametric verification on h_buffer_ was performed to check the influence on the device propagation parameters. First, the h_buffer_ was changed from 30 to 500 nm, and α and PL were evaluated, as shown in Figure 7. The propagation loss, shown in Figure 7a, increased until h_buffer_ = 90 nm, reaching α = 28010 dB/cm, although the PL = 1.55 μm. Figure 7b presents the PL behavior with the h_buffer_ increase. From h_buffer_ = 250 nm, the PL rose exponentially, related to the drop in propagation loss, which indicated weaker mode confinement, leading to lower sensitivity and a weak SPP effect [31].

To guarantee the LRSPP propagation, the propagation loss must be at least two to three times lower than the α_gold_ = 34299 dB/cm, with only the gold layer above the waveguide. For example, the h_buffer_ = 160 nm presented an α = 11770 dB/cm, which was 2.915 lower than α_gold_. Table 3 compiles some points of Figure 7.

As presented, the higher PL values indicated low attenuation, which directly influenced the sensitivity. Thus, the h_buffer_ = 300 nm was used in this work, to balance the propagation length and the propagation loss.

**Figure 8:**
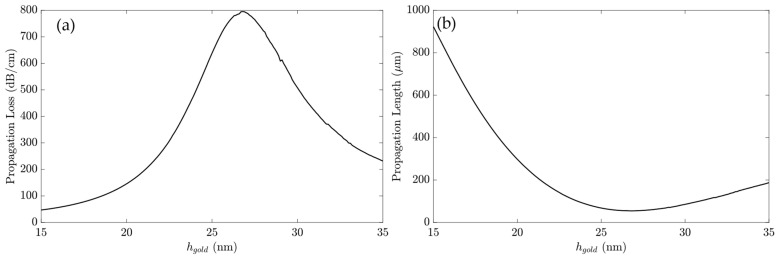
The Cytop layer h_buffer_ 300 nm. (**a**) α versus h_Au_ and (**b**) PL versus h_Au_.

Figure 8 shows the α and PL for the device with the Cytop layer with h_buffer_ = 300 nm and h_gold_ variation to investigate the influence of the gold thickness in the implemented h_buffer_. As shown in Figure 8a, the maximum propagation loss appeared at h_Au_ = 27 nm, with 790.8 dB/cm. The increase in α until h_Au_ = 27 nm agreed with the LRSPP theory, in which a thicker metal layer causes higher loss and tighter mode confinement around the gold layer [45]. Beyond that point, the propagation loss decreased since the SPP momentum match condition decreased, and the Si_3_N_4_ confined the field majority, reducing the hybrid mode propagation.

The addition of the Cytop layer increased the propagation length significantly, as shown in Figure 8b. At h_Au_ = 15 nm, the propagation length reached 922.8 μm, with a minimum at h_Au_ = 26.8 nm, with 54.61 μm. 

The propagation loss for the device with a Cytop layer at h_Au_ = 25 nm was 637.9 dB/cm. For example, the total loss for the device with a 70 μm long gold layer was 4.47 dB. Table 4 summarizes the waveguide performance parameters presented in Figure 6 and Figure 8.

With the increase in the h_Au_ height, the PL dropped significantly until h_gold_ = 27 nm. Beyond that point, the gold height reached the skin depth value for λ_0_ = 633 nm, causing the decoupling of the plasmonic modes. For that reason, the gold thickness h_Au_ = 25 nm, h_buffer_ 300nm, and L_Au_ = 70 μm were used for the following analysis. 

## 5. Results

**Figure 9:**
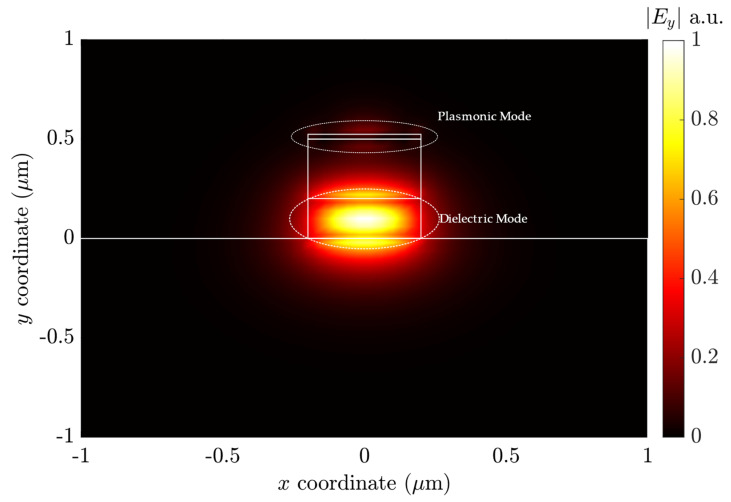
The Y-component of the electric field magnitude for the hybrid LRSPP mode.

The modal analysis performed by the method described in Section 3 presented the hybrid TM mode propagation, consisting of the fundamental dielectric and plasmonic modes, as shown in Figure 9. 

Figure 9 shows the electric field y-component for λ_0_ = 633 nm. The Si_3_N_4_ core contained the highest *E_y_* amplitude, with an evanescent field into the SiO_2_ and Cytop layers. At the gold layer, the absence of *E_y_* at the center and decaying fields on both sides confirmed the presence of the plasmonic mode. The structure presented a weak degenerate plasmonic corner mode [21], as seen similarly in [43]. Other modal analyses were performed for different wavelengths to analyze the hybrid mode behavior in the frequency spectrum 580 nm ≤ λ_0_ ≤ 680 nm. 

**Figure 10:**
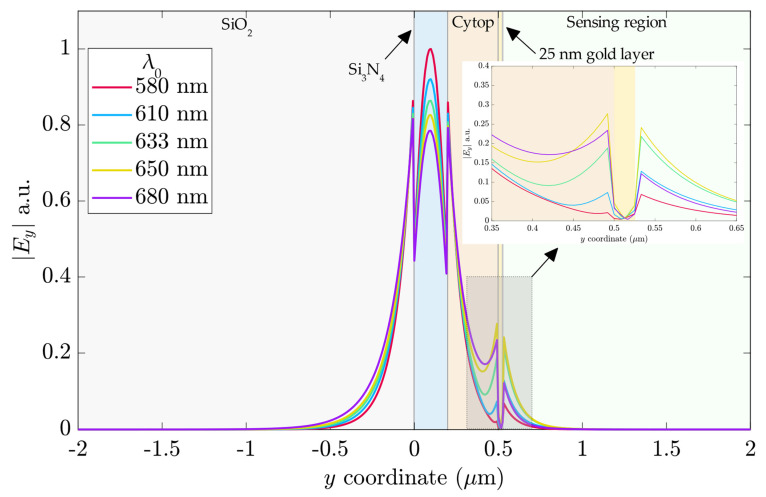
The electric field profile of the y-component at x = 0 μm. Inset: closeup of the gold layer region.

Figure 10 shows the *E_y_* profile of the hybrid mode at *x*-coordinate = 0 μm for five wavelengths, ranging from 580 nm to 680 nm, and *n_a_* = 1.33 in the sensing region. In the Si_3_N_4_ guiding region, the field profile was similar between the different wavelengths, with the highest amplitude for λ_0_ = 580 nm, while λ_0_ = 680 nm had the lowest field intensity.

The inset in Figure 10 shows the field profile of the plasmonic modes. For λ_0_ = 580 nm, the low field intensity at the gold layer led to a higher PL and a lower α, as Figure 10a,b present. The 610 nm and 680 nm wavelengths performed similarly at the sensing interface, although at the gold–Cytop interface, the electric field intensity was higher for λ_0_ = 680 nm. In the sensing region, the wavelengths interacting more with the analyte were 633 and 650 nm, which suggested more sensibility to n_a_ changes. However, the highest amplitude at the gold–Cytop interface for λ_0_ = 610 nm indicated a higher attenuation than λ_0_ = 633 nm.

**Figure 11:**
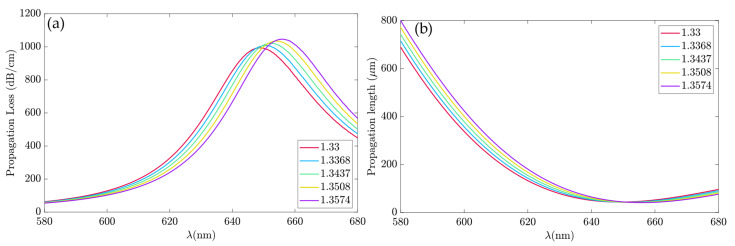
The propagation loss and propagation length spectrum for different glycerol concentrations. (**a**) Propagation loss. (**b**) Propagation length.

The PL and α correlated through Equations (8) and (9). The higher propagation loss led to a smaller propagation length, although the sensor sensitivity increased with higher losses. Figure 11a shows the proposed device propagation loss for five glycerol concentrations, as in Table 1, in the cited frequency spectrum. At λ_0_ = 580 nm, the α ranged from 63.23 to 54.46 dB/cm, as the n_a_ increased, while at λ_0_ = 633 nm, it went from 639.8 to 466.8 dB/cm. At the spectrum right edge, λ_0_ = 680 nm, the propagation loss ranged from 572 to 451.9 dB/cm.

The PL, shown in Figure 11b, decreased in propagation length from λ_0_ = 580 nm to λ_0_ = 650 nm. For *n_a_* = 1.33, PL = 690.5 μm, and for *n_a_* = 1.3574, it was PL = 801.1 μm, at λ_0_ = 580 nm. The PL reached the lowest values in the region between 640 and 660 μm. For example, at λ_0_ = 650 nm, the PL went from 42.97 μm for *n_a_* = 1.3437 to 44.52 μm for *n_a_* = 1.3574. 

**Figure 12:**
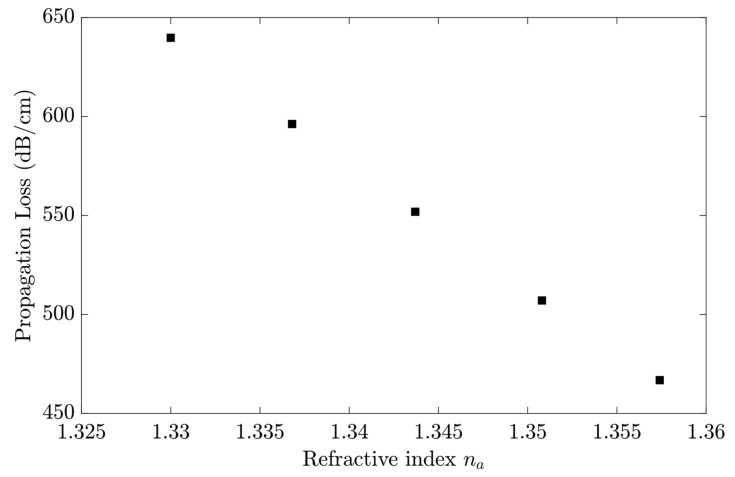
The LRSPP propagation loss shift versus the refractive index of different glycerol concentrations for λ_0_ = 633 nm.

The sensitivity to RI changes (∆*n_a_*) in the sensing region was evaluated using the shift in propagation loss (∆α) at a determined wavelength, as shown in Figure 12. The values are for the wavelength of 633 nm. Equation (10) estimates the bulk sensitivity in 6313.9 dB/cm/RIU.

(10) $$S=\frac{\Delta \alpha }{\Delta n_{a}}\;\left(dB/cm/RIU\right)$$

Another parameter to evaluate the sensor’s performance is the spectral sensitivity, calculated using Equation (11), ∆*n_a_* is the variation in the analyte’s refractive index, and ∆λ is the variation in the peak resonance wavelength.

(11) $$SS=\frac{\Delta \lambda }{\Delta n_{a}}\;\left(\frac{nm}{RIU}\right)$$

**Figure 13:**
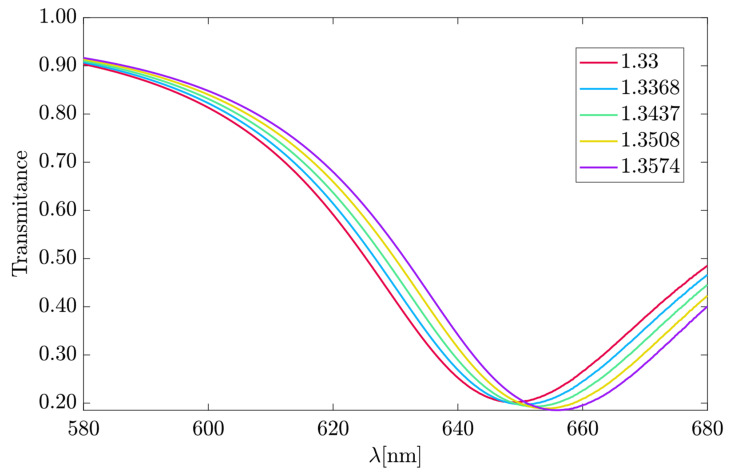
The transmittance spectrum of the LRSPP hybrid mode.

The transmittance (T = |t|²) spectrum in Figure 13 was obtained using the TMM explained in Section 3. The increase in the RI of the analyte caused a redshift in the T spectrum. As a result, the spectrum for each analyte had its minimum transmittance between 640 and 660 nm. For example, for *n_a_* = 1.33, the minimum was T = 0.2017, while for *n_a_* = 1.3574, it was T = 0.1853. The low transmittance in the 640 to 660 nm range was directly related to the highest values of the propagation loss (Figure 11a) in the same range.

As the structure dimensions were projected for λ_0_ = 633 nm, the SPP effect became stronger around λ_0_ = 610 nm until λ_0_ = 650 nm, for *n_a_* = 1.33, as seen in Figure 13 with the transmittance decrease. The electric field at the gold–dielectric interfaces increased in that region, as shown in Figure 10, indicating a higher attenuation. The linearity between 610 ≤ λ_0_ ≤ 650 nm followed the propagation loss behavior related to the extinction coefficient obtained by the FEM method described.

**Figure 14:**
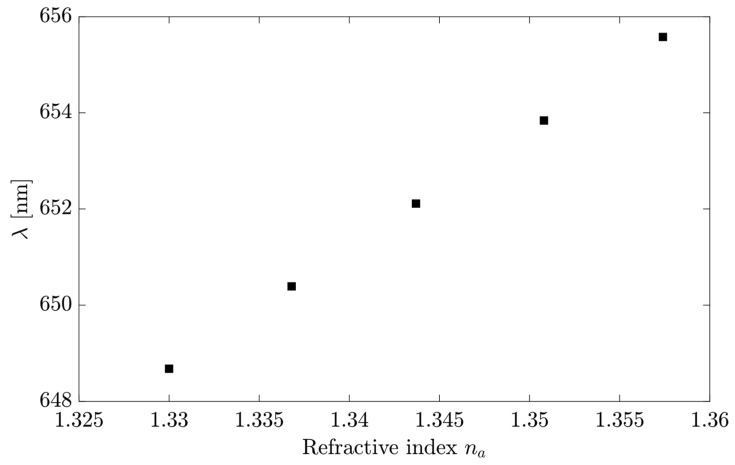
The LRSPP wavelength shift versus the refractive index of different glycerol concentrations for region II.

Equation (11) calculates the spectral sensitivity of 251.82 nm/RIU deduced from Figure 14. The wavelength values for each RI were referents of the minimal transmittance position in the frequency spectrum shown in Figure 13. 

Lastly, Table 5 shows a performance comparison between some photonic biosensors found in the literature and this work. Compared with similar structures, this work has a higher sensitivity, even with a more straightforward device configuration.

## 6. Discussion

This work presented a numerical study of an LRSPP waveguide RI sensor for glycerol concentrations. The device was simulated using a proper finite element method model and designed for a wavelength of 633 nm. The sensor structure was optimized for the waveguide width, buffer, and gold layer thickness. A comparison of the sensor performance with and without a Cytop buffer cladding was performed, showing the sensor improvement with the addition of the polymer layer. The frequency response ranging from 580 to 680 nm was determined using a TMM algorithm, which presented a redshift in the spectrum with the glycerol RI increase. The spectral sensitivity for a 70μm long sensor was about 252 nm/RIU, with a bulk sensitivity of 6313 dB/cm/RIU, at the wavelength of 633 nm. This sensor has the potential for POC applications because of its portability and simplicity of construction.

## Figures and Tables

**Table 1:** The refractive index for the glycerol solution in water.

Refractive Index n_a_	Glycerol Concentration (%)
1.3300	0
1.3368	5
1.3437	10
1.3508	15
1.3574	20

**Table 2:** Values of the Drude–Lorentz model.

i	f_i_	ω_i_ (rad/s)	Γ_i_ (rad/s)
0	0.760	0	8.05202 × 10^13^
1	0.024	6.30488 × 10^14^	3.66139 × 10^14^
2	0.010	1.26098 × 10^15^	5.24141 × 10^14^
3	0.071	4.51064 × 10^15^	1.32175 × 10^15^
4	0.601	6.53885 × 10^15^	3.78901 × 10^15^
5	4.384	2.02364 × 10^16^	3.36362 × 10^15^

**Table 3:** The propagation length and the propagation loss for h_buffer_ variation.

h_buffer_ (nm)	PL (μm)	α (dB/cm)	α_gold_/α
160	3.70	11770	2.915
200	8.49	5118	6.7
250	24.19	1795	19.1
300	68.09	637.8	53.78
350	189.50	229.2	149.67
400	522.10	83.18	412.37
450	1429	30.40	1128.3
500	3890	11.17	3070.6

**Table 4:** The plasmonic propagation parameters.

h_Au_ (nm)	PL (μm)	PL with Cytop (μm)	α (dB/cm)	α with Cytop (dB/cm)
15	3.753	922.80	11660	47.06
20	2.654	297.90	16360	145.80
25	1.266	68.08	34300	637.90
30	1.261	85.44	34430	508.30
35	1.551	197.30	28010	231.80

**Table 5:** Comparison of the performance metrics of selected optical biosensors.

Platform	λ_0_	RI Range	Sensitivity (RIU^−1^)	Reference
Bragg grating	1550 nm	1.3334–1.3526	106 nm	[46]
Bragg grating	1500–1550 nm	-	182 nm	[47]
Straight LRSPP	850 nm	1.562–1.82	1960 dB/cm	[31]
LSPR	700–735 nm	1.334–1.35	248.3 nm	[48]
This work	580–680 nm	1.33–1.3475	251.82 nm 6131.9 dB/cm	-

## Data Availability

Not applicable.
